# Improving Census Transform by High-Pass with Haar Wavelet Transform and Edge Detection

**DOI:** 10.3390/s20092537

**Published:** 2020-04-29

**Authors:** Jiun-Jian Liaw, Chuan-Pin Lu, Yung-Fa Huang, Yu-Hsien Liao, Shih-Cian Huang

**Affiliations:** 1 Department of Information and Communication Engineering, Chaoyang University of Technology, Taichung 413310, Taiwan; jjliaw@cyut.edu.tw (J.-J.L.); yfahuang@cyut.edu.tw (Y.-F.H.); s10630608@gm.cyut.edu.tw (Y.-H.L.); s10230612@gm.cyut.edu.tw (S.-C.H.); 2 Department of Information Technology, Meiho University, Pingtung 912009, Taiwan

**Keywords:** census transform, sparse census transform, disparity, stereo vision

## Abstract

One of the common methods for measuring distance is to use a camera and image processing algorithm, such as an eye and brain. Mechanical stereo vision uses two cameras to shoot the same object and analyzes the disparity of the stereo vision. One of the most robust methods to calculate disparity is the well-known census transform, which has the problem of conversion window selection. In this paper, three methods are proposed to improve the performance of the census transform. The first one uses a low-pass band of the wavelet to reduce the computation loading and a high-pass band of the wavelet to modify the disparity. The main idea of the second method is the adaptive size selection of the conversion window by edge information. The third proposed method is to apply the adaptive window size to the previous sparse census transform. In the experiments, two indexes, percentage of bad matching pixels (PoBMP) and root mean squared (RMS), are used to evaluate the performance with the known ground truth data. According to the results, the computation required can be reduced by the multiresolution feature of the wavelet transform. The accuracy is also improved with the modified disparity processing. Compared with previous methods, the number of operation points is reduced by the proposed adaptive window size method.

## 1. Introduction

The development of automatic equipment has always been one of the focuses in the field of computer science. The features of automatic equipment are its self-moving and exploring characteristics that can be used to reduce the risk of participation of personnel. In many automated devices, the distance of the device from the object in the surrounding environment is an important parameter, such as using vision to avoid obstacles. In modern automated or intelligent devices, the distance from the object is an important indicator. The corresponding action can be performed by determining the distance, whether this is using a robot arm to grab the object [1], automatic car driving to determine the road condition [2,3], or using a robot that can self-plan a path to shuttle through the environment [4]. All of these studies show that measuring distance is an essential part of achieving automation.

The methods of measuring distance are roughly divided into two types. The first one uses ray or waves as the direct measurement method [5]. These methods use laser, infrared, or ultrasonic means to shoot toward the object and simultaneously record the time of transmission and the time of receiving the reflection. The distance of the object can be calculated by the time difference between the transmission and reception. The second method uses a camera and image processing or machine vision technologies [6]. These methods use a camera to capture an image of an object, and then analyze the pixels in the image and measure the distance of the object. This type of method is mainly divided into two types: monocular vision with additional reference objects and two-eye stereoscopic vision.

The method of using ray or sound waves is more accurate and faster than the method of using a camera with an algorithm, but the equipment is expensive and the application is not easy to popularize. In recent years, in the case of the popularity of digital cameras, the technology for obtaining distance by image processing schemes has gradually received attention. Furthermore, as the advancement of the computer has improved the speed of this in recent years, the performance of two-eye stereoscopic processing has also improved. Even when using an accessible style camera, the stereo vision system can also be applied to measure the target distance [7].

In the steps of using image processing or machine vision technology to measure distance, the most-time consuming loading step is matching. The matching includes object recognition or feature extraction [8]. The problems of object recognition or feature extraction can be divided into hardware and algorithm domains. In the hardware methods, a field-programmable gate array can be used to implement the spike-based method [9]. The circuit can be also designed to achieve the efficiency of low power consumption by combining an active continuous-time front-end logarithmic photoreceptor [10]. In the algorithm methods, the visual information (such as an image) can be calculated by the address-event-representation [11] or by constructing stereo vision with cameras [12]. However, the devices for matching are more expensive than consumer cameras. It is easier and more effective when we use the algorithms with general cameras to solve the matching problem.

Most common cameras record two-dimensional images, with only the horizontal axis and the vertical axis. Since the camera takes images only in two dimensions, the distance measurement function that can be achieved is limited. To solve this problem, scholars have proposed mechanical stereo vision. This uses two cameras to shoot the same object from different positions and analyze the distance between the camera and the object by the algorithms of image processing or machine vision. The axis perpendicular to the image plane and the two axes of the image plane constitute the three-dimensional relationship between the camera and the object [12].

**Figure 1:**
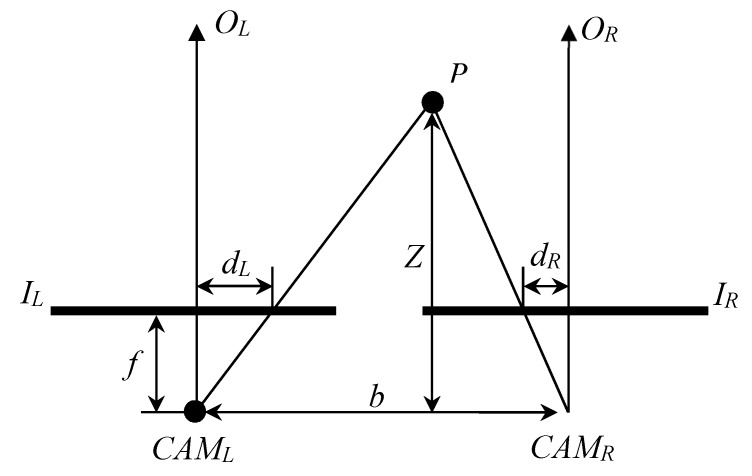
Schematic diagram of a three-dimensional model with disparity.

When we use two cameras to observe the same object, the positions of the object on the two images will be slightly different. This difference is called disparity. A simple two-eye stereo vision model is shown in Figure 1, where *P* is the target, *CAM_L_* and *CAM_R_* are the two cameras, *b* is the distance between two cameras, *f* is the focal length, *I_L_* and *I_R_* are the imaging planes, *d_L_* and *d_R_* are the distances between targets on the image planes and the centers of the images, *O_L_* and *O_R_* represent the center lines of the lens, and *Z* is the distance we are looking for. We can see that *Z* can be calculated by the relationship of similar triangles [13]:

(1) $$\frac{b}{Z}=\frac{b-\left(d_{L}+d_{R}\right)}{Z-f}=\frac{d_{L}+d_{R}}{f}$$

The disparity is denoted as *d_L_* + *d_R_*, and it is the amount of horizontal displacement that is produced by the same object that is imaged by two cameras. Since both *f* and *b* are known, it can be seen that it is quite important to obtain the disparity in the stereo vision system. The key to obtaining the disparity is matching the same object in the two images [14,15].

As shown in the above description, the method for obtaining three-dimensional information by the mechanical stereo vision system is to analyze and obtain the disparity between the two images. Census transform (CT) is one of the most robust algorithms for calculating the disparity of two images [16]. When we use CT, the size of the conversion window directly affects the computational load and accuracy. In a previous study, it was confirmed that a larger conversion window makes the result of object matching more accurate [17,18]. When the conversion window is larger than a certain size, the matching performance is not as significant as the window becomes larger. However, an oversized window not only consumes computational resources but also makes too many errors in matching. Therefore, the size of the window in CT is one of the important keys to determining the performance [19].

The calculation of each pixel by CT requires a large computational load and memory requirements. This makes it difficult for CT to be applied in real-time systems. Since the object in the image is bound by its edge, which is a sudden change in intensity, the edge (the high-frequency information in the image) is an important image feature [20]. This edge detection and the high-frequency information method are very important parts of image processing. These have also been applied in many real applications, such as oil spill detection for offshore drilling platforms [21], vehicle license plate location identification [22], pedestrian detection [23], and gesture recognition [24]. In the stereo vision system, a boundary can be used to identify whether the region is flat or has texture. The boundaries in the image can be obtained by gradients. Changing the length and width of the window according to the vertical and horizontal gradients can be used to reduce the bad matching of CT [25]. However, the quality of the disparity and operation loading are not discussed. The disparity quality can be improved by matching with a variable window and *p* post-processing with sub-pixel interpolation after CT [26]. This method does not adjust the window size when performing CT. However, using the sub-band of the high-frequency (such as edge) to improve the performance of CT is one of the feasible methods. Wavelet transform is a multi-resolution analysis method. The image data can be transformed into different sub-bands according to the defined wavelet [17]. The Haar wavelet is a well-known method to analyze the frequency information from sub-bands [27].

In this paper, two methods are proposed to improve the performance of CT using edge information. The first method is named census transform with Haar wavelet (CTHW) and uses edge information that is extracted by a wavelet. Since the edge information provides more accurate object information, the high-passed data is used to modify the disparity. The second method is called an adaptive window census transform (AWCT). The AWCT can determine whether the boundary of the window is increased or not when the window is enlarged. The increased rate of the boundary pixels in the window is used to determine the suitable window size. Moreover, since the sparse census transforms can be used to enhance the CT’s performance by the designed mapping positions [28]; we also applied sparse census transforms to AWCT. AWCT and adaptive window sparse census transform (AWSCT) are applied to avoid using the oversized window and improving the performance.

## 2. Related Methods

### 2.1. The CT Algorithm

CT converts the grayscale intensity represented by each pixel in the grayscale image into the grayscale intensity relationship of each pixel to the neighbor pixels. The relationship can be treated as a feature of the pixel and used to find the two most similar features in the left and right images by the Hamming distance. The positions of the most similar points can be used to compute the disparity by Equation (1).

CT is defined as an order in a local neighborhood (conversion window) by comparing the relationship between the center pixel and the neighborhood pixels. The relationship between the center point (denoted as *p*) and the neighbor point (denoted as *p*’) can be described by the conversion function [16,29]:

(2) $$\xi (p,p\prime )=\{\begin{array}{c} 1,\\ 0, \end{array}\begin{array}{c} \;if\;I(p)>I(p\prime )\\ \;otherwise \end{array}$$

where *I*() is the intensity of the pixel. A conversion window is defined to select neighbor pixels. In Equation (2), *p* is located at the center of the window, and the other pixels located in this window are selected to be *p*’ in turn. Usually, the shape of the converted window is square, and the size is user-defined. The CT at the pixel *p* can be written as

(3) $$C(p_{xy})=\underset{p_{ij}\in w}{\overset{}{⊗}}\xi (I(p_{xy}),I(p_{ij}))$$

where ⊗ is the concatenation operator and *w* is the conversion window. In the stereo vision with CT, two images (such as *I_L_* and *I_R_* in Figure 1) are transformed by CT and hamming distance is applied to obtain the disparity between two transformed images. The disparity can be determined by using winner-takes-all to find the minimum value among all possible disparity values [30].

### 2.2. The Haar Wavelet

The wavelet transform converts signals into small waves and performs signal processing and signal analysis with multi-resolution. It is widely used in compression, transmission and image analysis [31]. The Haar wavelet was proposed by Alfréd Haar in 1909 [27]. It is the earliest proposed and simplest type of wavelet transforms [32]. With two pixels (*p*_1_ and *p*_2_) in the image, the Haar wavelet can be implemented to the low-band and high-band by

(4) $$\mathrm{Low}\;\mathrm{band}=\left(p_{1}+p_{2}\right)/\sqrt{2}$$

and

(5) $$\mathrm{High}\;\mathrm{band}=\left(p_{1}-p_{2}\right)/\sqrt{2}$$

respectively. In practice, the Haar wavelet can be described as a transformation matrix [32]:

(6) $$Haar=\frac{\sqrt{2}}{2}\left[\begin{array}{cc} 1 & 1\\ 1 & -1 \end{array}\right]$$

We can see that the image is high-passed and low-passed in the *x*-direction with down-sampling. The result obtained is also high-passed and low-passed in the *y*-direction with down-sampling. Finally, we obtain four sub-bands of LL (horizontal low-band and vertical low-band), LH (horizontal low-band and vertical high-band), HL (horizontal high-band and vertical low-band), and HH (horizontal high-band and vertical high-band).

### 2.3. Edge Detection

The boundary information can be extracted by edge detection and regarded as a result of high-pass filtering. The result of edge detection is mainly used to highlight whether the image is an area where the pixel changes significantly. In this study, the boundary information is used to classify and determine the complexity in the vicinity of the pixel. If the gray scale intensity changes significantly, a smaller conversion window can be used; otherwise, if the gray scale intensity of the area near the pixel does not change significantly, a larger conversion window must be used. In this paper, the Canny edge detection method is used for boundary detection [33,34]. First, the noise must be filtered by a two-dimensional Gaussian filter. The Gaussian function can be described as

(7) $$G\left(x,y\right){=e}^{-\frac{x^{2}{+y}^{2}}{2\sigma^{2}}}$$

where σ is the variance of the Gaussian function and it can be regarded as a smoothing factor in the filtering. The Gaussian function and the image can be computed by convolution to obtain the amount of change in the *x* and *y* directions, which are denoted as *g_x_* and *g_y_*. The gradient based on the pixel value in the image can be expressed as the gradient magnitude and the gradient direction, by

(8) $$I_{gm}\left(x,y\right)=\sqrt{g_{x}^{2}+g_{y}^{2}}$$

and

(9) $$I_{g\theta }\left(x,y\right)=\tan^{-1}\left(\frac{g_{y}}{g_{x}}\right)$$

respectively. Since the edge can be described as the gradient, the boundary points can be detected as the larger gradient magnitude on the gradient direction. We compare the pixels on the gradient direction with pixels not on the gradient direction. If the value of a pixel on the direction is larger than the value of pixel not on the direction, the pixel is regarded as a boundary point; otherwise, it is a non-boundary point.

## 3. Proposed Methods

### 3.1. Census Transform with Haar Wavelet (CTHW)

**Figure 2:**
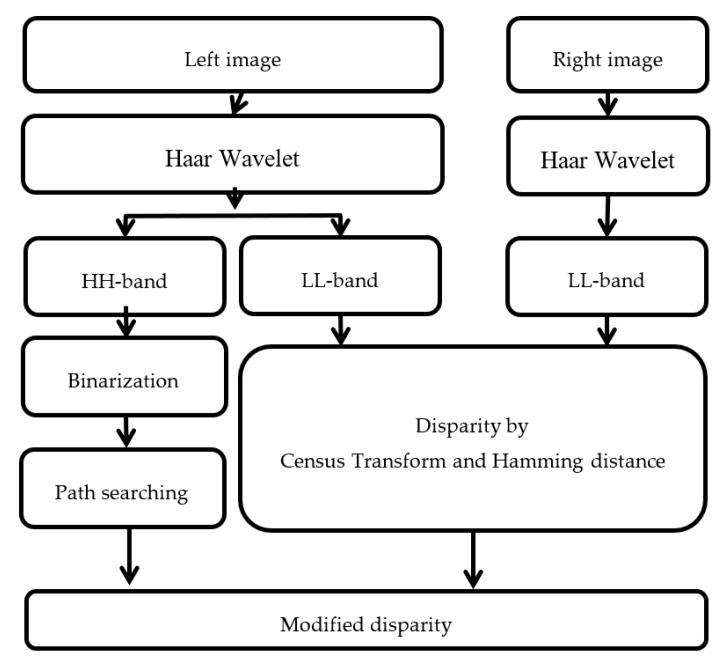
The flowchart of the census transform with Haar Wavelet.

In this section, the multi-resolution of the image with a Haar wavelet is used to reduce the computational time of the census transform (CT) and Hamming distance operations. The flow of this method is shown in Figure 2. First, the left and right view images are input. For both images, Haar wavelets are performed to obtain the frequency domain data of LL, HL, LH, and HH-bands. The outputs of LL-bands are converted by CT and the disparity calculated using the Hamming distance. The output of the HH-band from the left image is performed by path searching after binarization. The stop point of the path searching is determined by the high values in the HH-band. The disparity is modified to find the largest disparity that appears in the search paths.

**Figure 3:**
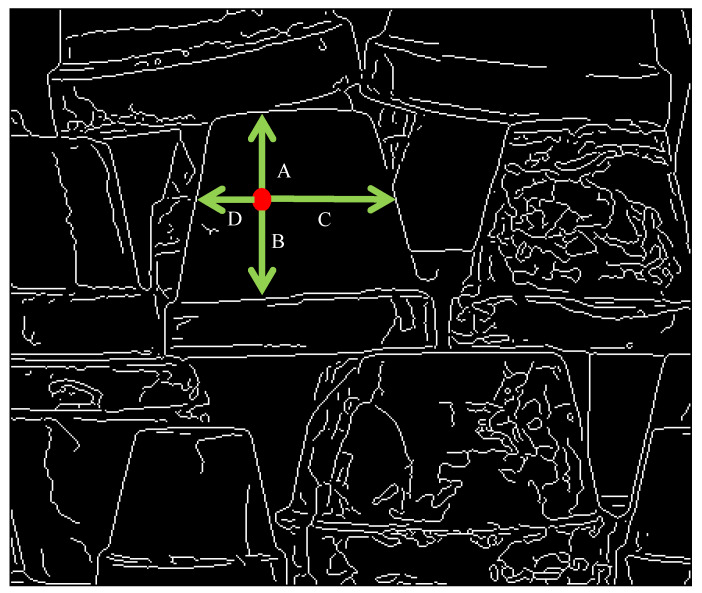
Example of the path searching of a (red) point.

Since the size of the LL-band is smaller than the original image, one main idea of this method is to use the LL-bands of original images to reduce the computational load of CT. Moreover, since the down-sampled image may result in errors, another main idea is to use the HH-band of one original image to modify the disparity. The path searches of the pixel in the HH-band are in four directions: up, down, right, and left. An example of an HH-band image is shown in Figure 3 and a point is colored in red. Since the mechanical stereo vision is applied by two horizontal cameras, the horizontal direction is the main searching path. In order to reduce the effect of large areas without borders, the vertical direction is added to the searching paths. The four searching paths are from the red point to four directions, which are denoted as A, B, C, and D with green arrows. Each searching path is stopped when the path arrives at the edge (white point). The corresponding disparity values (the output of CT with Hamming distance) on the paths are recorded and counted. The modified disparity is set as the disparity value with the largest number.

### 3.2. Adaptive Window Census Transform (AWCT)

The size of the conversion window will affect the computational load and accuracy when we use CT. The larger the conversion window, the more accurate the results, but this also consumes the computational resources. In this section, the proposed method, AWCT, changes the selection of the conversion window size. The conversion window size changes from a fixed size of all pixels to an adaptive size by the boundary around the pixel. The edge information is used to select the window size.

**Figure 4:**
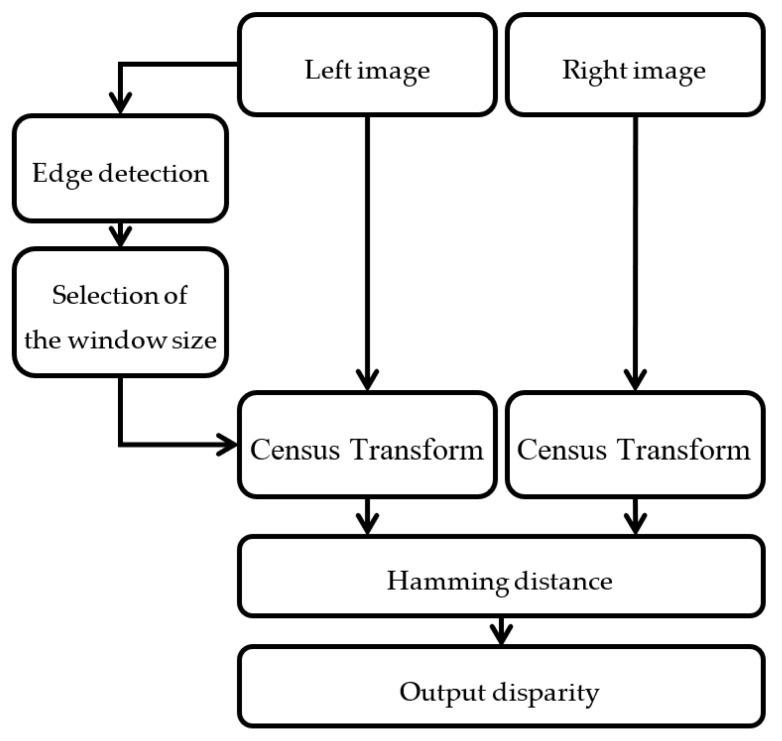
The flowchart of the adaptive window census transform (AWCT).

The flow chart of AWCT is shown in Figure 4. First, the edge information can be obtained from the left view image by edge detection. The conversion window size of each point can be determined by the edge information. The selected window sizes are applied for CT, and the disparity can be computed through a Hamming distance computation.

The conversion window sizes can be 3 × 3, 5 × 5, 7 × 7, …, 21 × 21. After the edge detection, each point will be the center of the windows in turn, and we count the number of edge points in each window. We also count the proportion of the number of edge points in the window. The window size ranges from small to large, and the size is selected when the window contains edge points. We divide the window size selection into two types. The first type is no edge, in which there are no edge points in the window. The image texture representation in this area is unclear, even without texture. The largest window size is used in this type. The other type is one in which the edge points are in the window. In this type, we record the proportion of edge points in the window when the window changes from small to large. We record it as one-time negative growth when the window size increases by one level and the ratio decreases. The window size will be selected when we have negative growth *N* consecutive times. In this study, the value of *N* is set to 5 through experience. This value can be set by the user for different cases.

**Figure 5:**
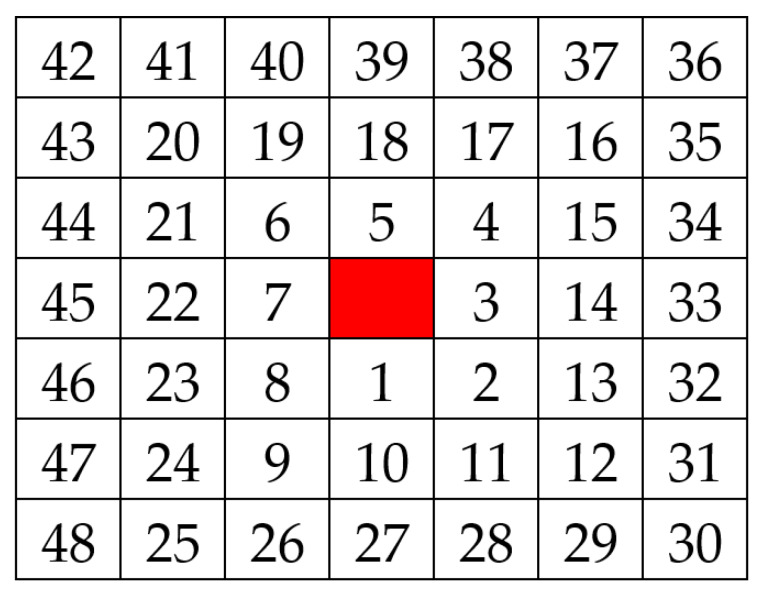
The order of pixel calculation (with a 7 × 7 window size).

Since the conversion window size is adaptive, the used window size of each pixel may be different. The smaller windows size is selected to compute the hamming distance when the sizes are different. In order to make the comparison of hamming distance reasonable, the calculation order of pixels is counter-clockwise from the center outward. An example of the pixels’ order is shown in Figure 5. Even if the two windows are different in size, this order will make the relative positions of the pixels the same. The comparison of Hamming distance is up to the length of the small window. This allows the hamming distance to be used in the same window size.

### 3.3. Adaptive Window Sparse Census Transform (AWSCT)

**Figure 6:**
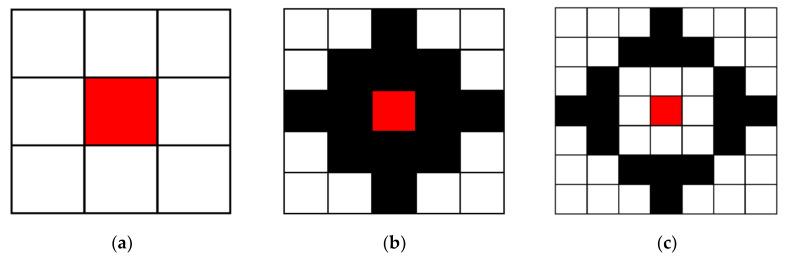

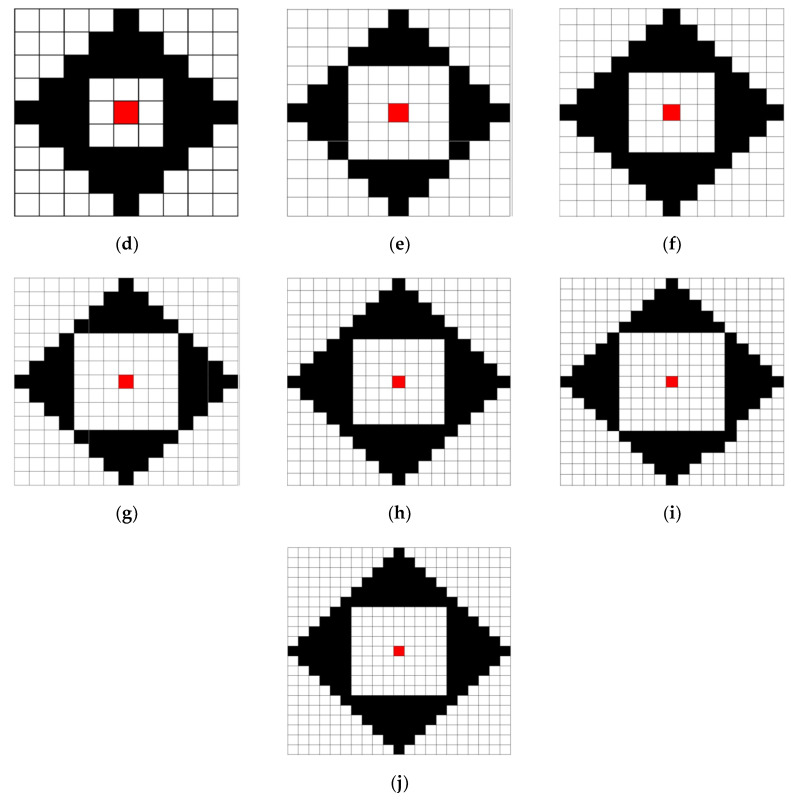
The selected points in the windows with a sparse census transform. (**a**) AWSCT 3 × 3, (**b**) AWSCT 5 × 5, (**c**) AWSCT 7 × 7, (**d**) AWSCT 9 × 9, (**e**) AWSCT 11 × 11, (**f**) AWSCT 13 × 13, (**g**) AWSCT 15 × 15, (**h**) AWSCT 17 × 17, (**i**) AWSCT 19 × 19, and (**j**) AWSCT 21 × 21.

According to Equation (3), we can see that the number of points to be computed will increase as the window size increases. Since some points may be ignored to reduce the operation times of the computer, the sub-set of points of the conversion window is applied to determine which points are calculated for CT. This modified method is called a sparse census transform, and it is defined as [28]

(10) $$C(p_{xy})={\underset{p_{ij}\in w}{\overset{}{⊗}}}_{s}\xi (I(p_{xy}),I(p_{ij}))$$

where *w_s_* is the sub-set of points of the conversion window. We can see that the sparse census transform takes a part of the points to convert instead of all the points in the window. According to the results of the sparse census transform [28], the neighbor points are selected to be symmetric. The 16-points are selected with a 7 × 7 window as shown in Figure 6c, which maximizes the performance. In this paper, the same pattern of Figure 6c is used and expanded for the selected points to the different windows. The selected points with different windows are shown in Figure 6. In this section, the sparse census transform is combined with AWCT. The flowchart of AWSCT is the same as AWCT (Figure 4), but the CT is changed to SCT (sparse census transform).

## 4. Experiments and Results

The results were compared with the ground truth data by PoBMP (percentage of bad matching pixels) and RMS (root mean squared) [35]. The PoBMP is defined by

(11) $$\mathrm{PoBMP}\;=\frac{1}{N}\sum_{\left(x,\;y\right)}{\;(|d}_{C}\left(x,\;y\right)-d_{T}{(x,\;y)\;|)\;>\;\delta }_{d}$$

where *d_C_* is the disparity with the proposed method, *d_T_* is the disparity with the ground truth and $\delta_{d}$ is the allowable error which is set as 3 in this paper. The RMS can be obtained by

(12) $$\mathrm{RMS}\;={\left(\;\frac{1}{N}\;\sum_{\left(x,y\right)}{|\;d}_{C}\left(x,y\right)-d_{T}{\left(x,y)\;\right|}^{2}\right)}^{\frac{1}{2}}$$

**Figure 7:**
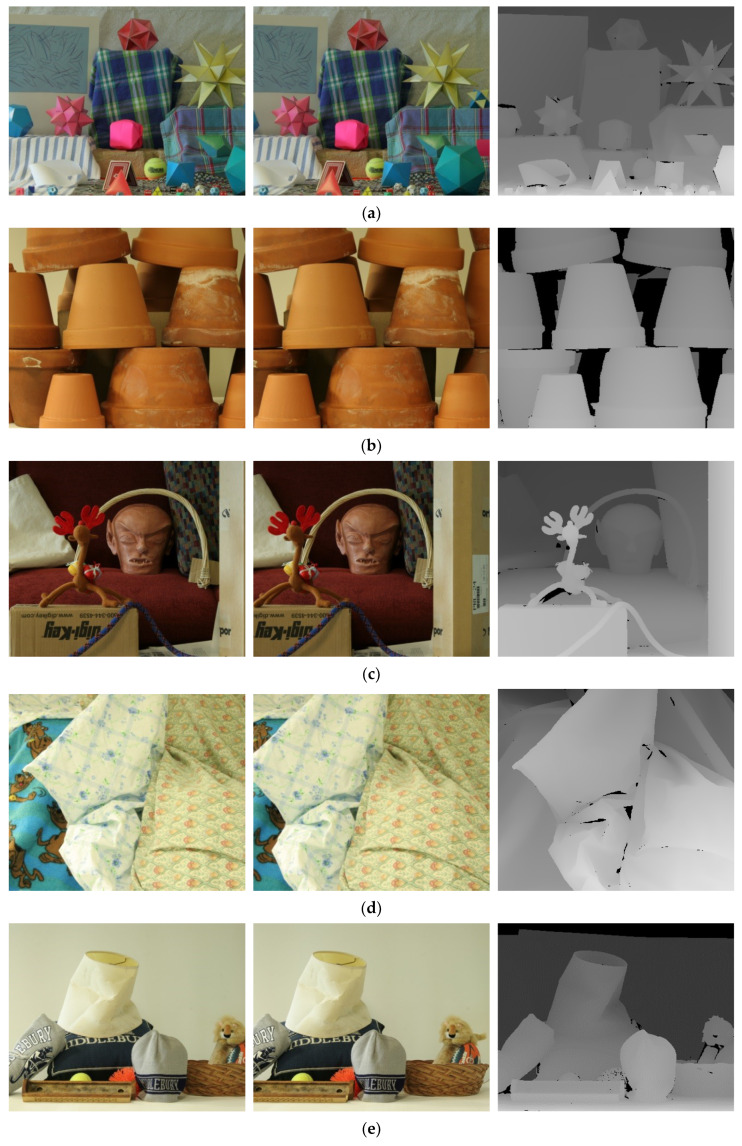

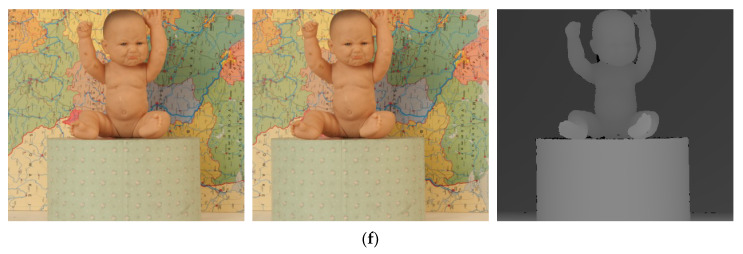
The experimental images: sequentially, the right image, left image and ground truth. (**a**) Moebius, (**b**) Flowerpots, (**c**) Reindeer, (**d**) Cloth2, (**e**) Midd1, and (**f**) Baby1.

Six images (Moebius, Flowerpots, Reindeer, Cloth2, Midd1, and Baby1), which were provided by Middlebury Stereo Datasets [36], were used to show the performances of the proposed methods. These six images and their ground truth are shown in Figure 7.

### 4.1. Results of CTHW

**Figure 8:**
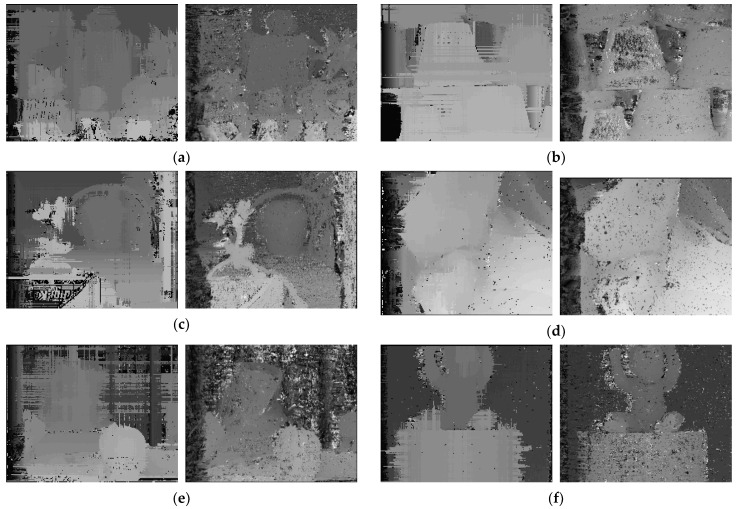
The results, sequentially, of CTHW and CT. (**a**) Moebius, (**b**) Flowerpots, (**c**) Reindeer, (**d**) Cloth2, (**e**) Midd1, and (**f**) Baby1.

The disparity results of six images by CTHW and CT with a 21 × 21 window size are shown in Figure 8, and the PoBMP results are shown in Table 1. According to the results, we can see that we obtained a better PoBMP with the proposed CTHW. Especially in the case of small conversion windows, such as 3 × 3 and 5 × 5, the PoBMP was less than 10% lower than the CT. In some exceptional cases—for example, Reindeer and Cloth2 with 13 × 13 window sizes—although the PoBMP of CTHW was higher than the PoBMP of CT, the accuracy of CTHW was still higher with a small window size. In the disparity results, the black points represent unknown disparity. We can see that the disparity images of CTHW was better than that of CT because there were significantly fewer black points. The results show that CTHW obtained better disparity results.

### 4.2. Results of AWCT

**Figure 9:**
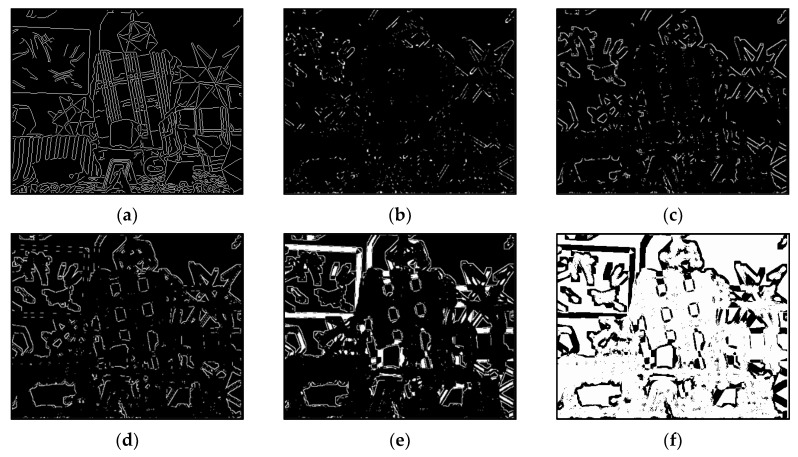
The results of Moebius by AWCT. (**a**) Edge information and schematic diagrams of the biggest window size in (**b**) 13 × 13, (**c**) 15 × 15, (**d**) 17 × 17, (**e**) 19 × 19, and (**f**) 21 × 21.

**Figure 10:**
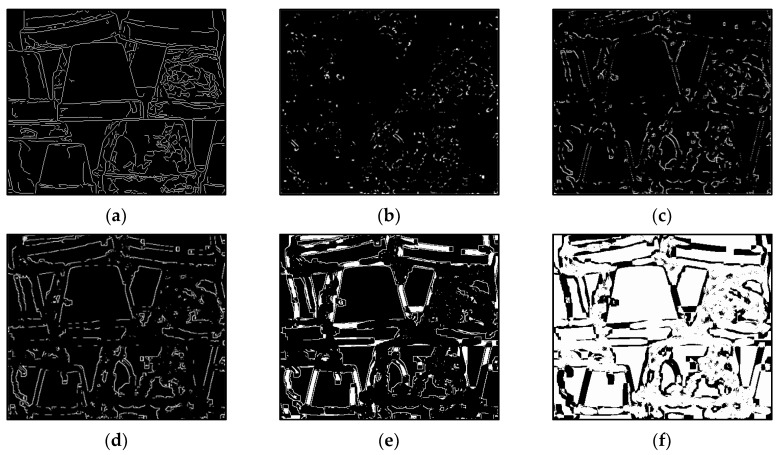
The results of Flowerpots by AWCT. (**a**) Edge information and schematic diagrams of the biggest window size in (**b**) 13 × 13, (**c**) 15 × 15, (**d**) 17 × 17, (**e**) 19 × 19, and (**f**) 21 × 21.

**Figure 11:**
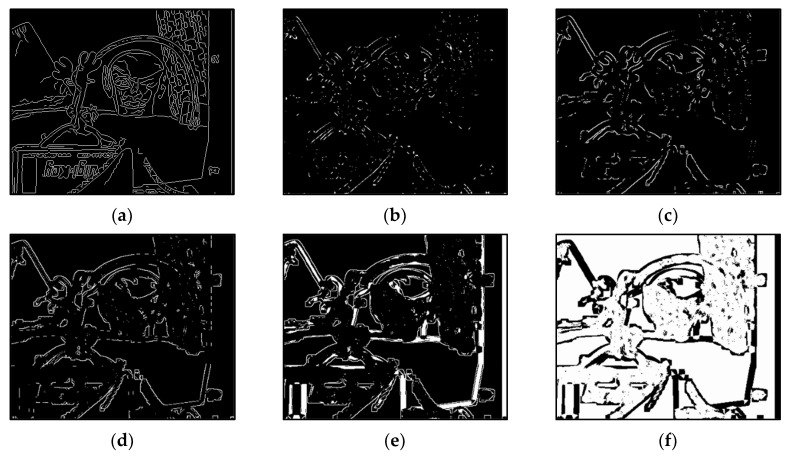
The results of Reindeer by AWCT. (**a**) Edge information and schematic diagrams of the biggest window size in (**b**) 13 × 13, (**c**) 15 × 15, (**d**) 17 × 17, (**e**) 19 × 19, and (**f**) 21 × 21.

**Figure 12:**
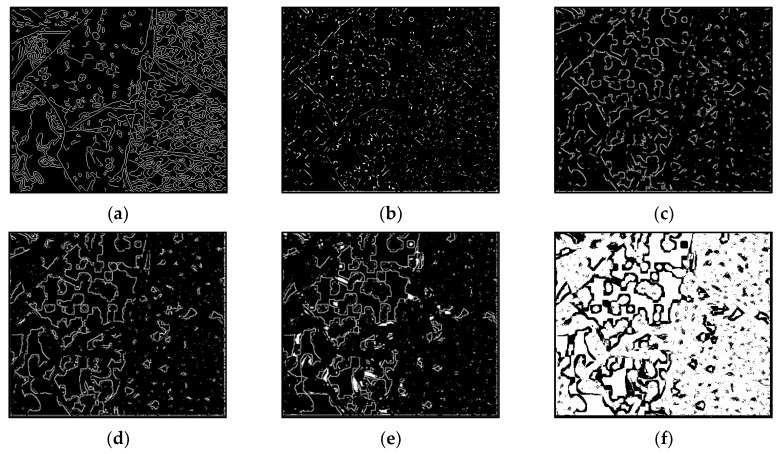
The results of Cloth2 by AWCT. (**a**) Edge information and schematic diagrams of the biggest window size in (**b**) 13 × 13, (**c**) 15 × 15, (**d**) 17 × 17, (**e**) 19 × 19, and (**f**) 21 × 21.

**Figure 13:**
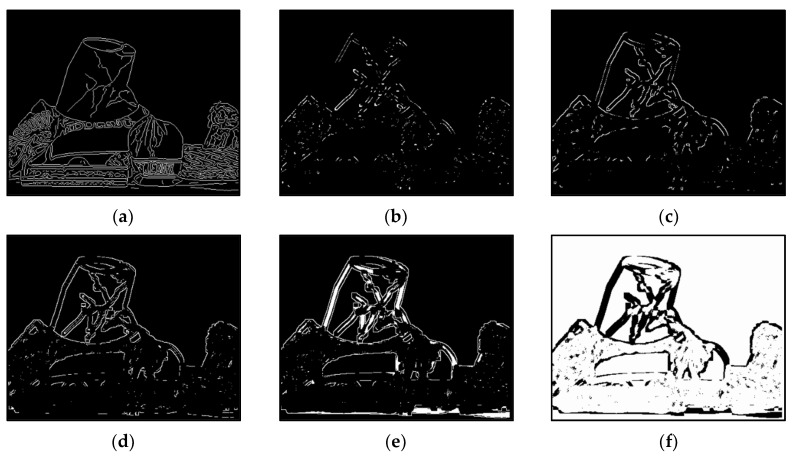
The results of Midd1 by AWCT. (**a**) Edge information and schematic diagrams of the biggest window size in (**b**) 13 × 13, (**c**) 15 × 15, (**d**) 17 × 17, (**e**) 19 × 19, and (**f**) 21 × 21.

**Figure 14:**
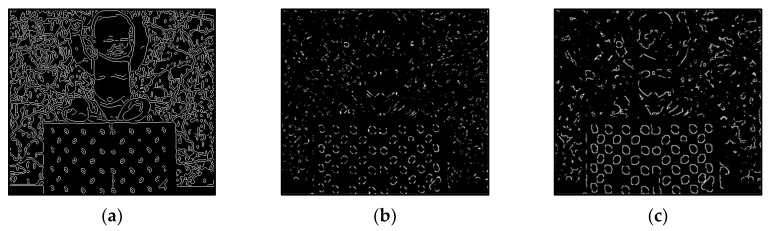

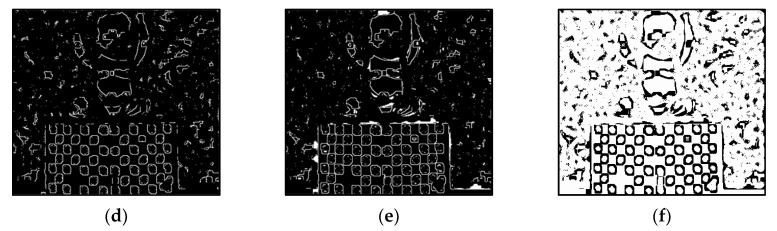
The results of Baby1 by AWCT. (**a**) Edge information and schematic diagrams of the biggest window size in (**b**) 13 × 13, (**c**) 15 × 15, (**d**) 17 × 17, (**e**) 19 × 19, and (**f**) 21 × 21.

**Figure 15:**
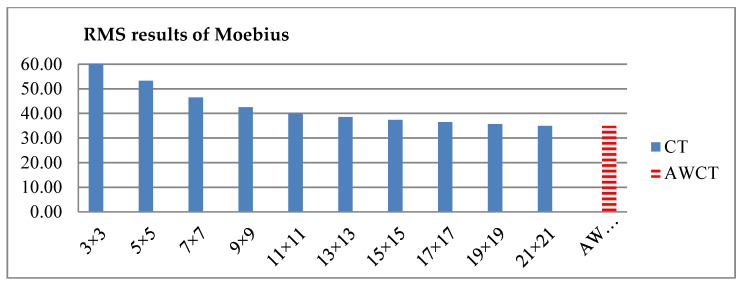
RMS comparison results of Moebius between AWCT and CT.

**Figure 16:**
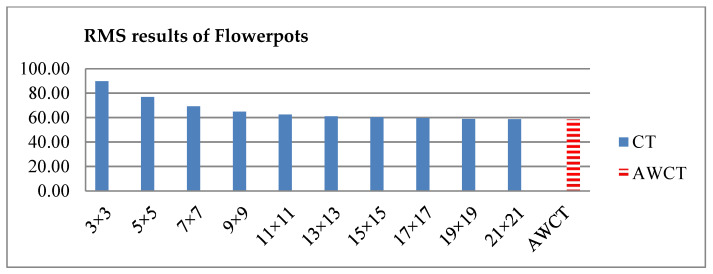
RMS comparison results of Flowerpots between AWCT and CT.

**Figure 17:**
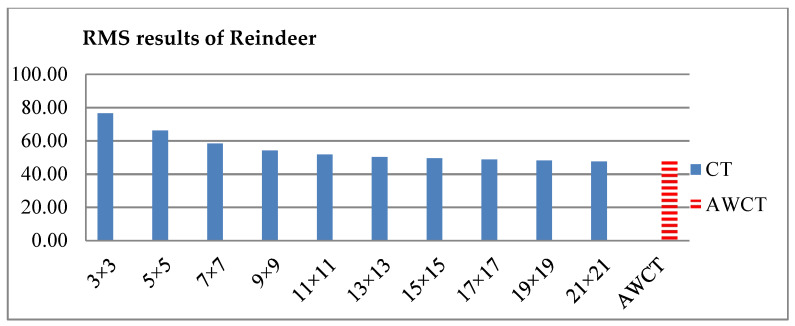
RMS comparison results of Reindeer between AWCT and CT.

**Figure 18:**
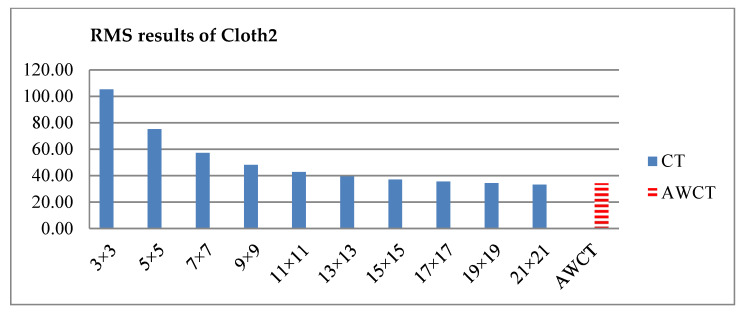
RMS comparison results of Cloth2 between AWCT and CT.

**Figure 19:**
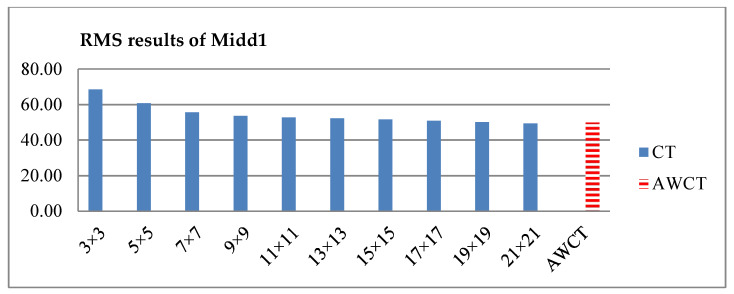
RMS comparison results of Midd1 between AWCT and CT.

**Figure 20:**
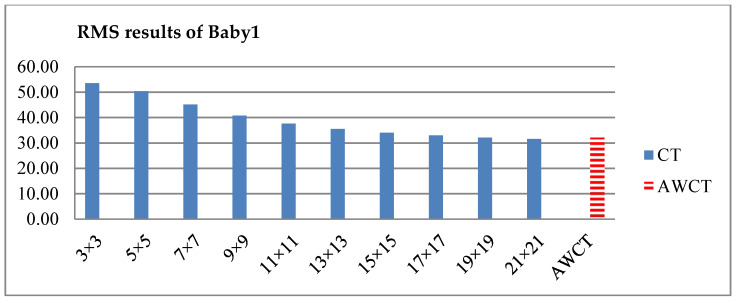
RMS comparison results of Baby1 between AWCT and CT.

The results of Moebius by AWCT are shown in Figure 9. The edge detection result is shown in Figure 9a. The main idea of the AWCT is that the windows can be adapted. The worst option is to select the largest window size. In order to show the worst case for the size of each window, the pixels which are adapted to the largest windows size in 13 × 13, 15 × 15, 17 × 17, 19 × 19, and 21 × 21 are set as white and shown in Figure 9b–f, respectively. We can observe that when using a 13 × 13 and 15 × 15 window size, not all edge areas are applied to the largest window. At a 17 × 17 and 19 × 19 window size, the largest window was used for almost all edge points. Similar experimental results of Flowerpots, Reindeer, Cloth2, Midd1 and Baby1 are also shown in Figure 10, Figure 11, Figure 12, Figure 13 and Figure 14, respectively. The results of six images with RMS are shown in Figure 15, Figure 16, Figure 17, Figure 18, Figure 19 and Figure 20, respectively. These results show that AWCT’s RMS is equivalent to the results of the largest windows (7 × 17, 19 × 19 or 21 × 21) with CT. The detailed results of AWCT and the results of CT with the largest window are listed in Table 2. According to the results, we can see that when we use the windows sizes with 13 × 13, 15 × 15, 17 × 17, or 19 × 19, the number used of the largest window is significantly less. This means that the AWCT can effectively adjust the window size and reduce the number of the largest windows. The results also show that the accuracy (PoBMP and RMS) of AWCT is similar to that of CT, but the reduction ratio of the operation number of calculation points (total pixels are calculated in Equation (3)) is about 4%–7%.

### 4.3. Results of AWSCT

The results of AWSCT and the results of SCT with the largest window are listed in Table 3. The experimental results show that the accuracies (PoBMP and RMS) of the two methods are similar, but the proposed AWSCT is better within the terms of operational requirements. This means that AWSCT can use fewer computing resources to achieve the same accuracy.

### 4.4. Discussion of Results

The results of CTHW show that using wavelet’s high-frequency band with path searching to modify disparity can effectively reduce PoBMP. This is because most bad matching is replaced by other disparities, but the modified disparity may not be accurate. Since the main problem of using CT is conversion window selection, it is easy to understand that CTHW (without adjusting the window) is not better in RMS and operation reduction. Based on high-frequency technology, we propose an AWCT method that uses edges to adjust the window size. The results show that AWCT’s quality (PoBMP and RMS) is acceptable with a reduction of 4%–7% operation. Applying the sparse concept to AWCT can also reduce the operation by 5%–9% compared to SCT.

## 5. Conclusions

One of the well-known methods for obtaining disparity is called CT. We discussed the key problem of CT, which is the size of the conversion window. The larger the conversion window, the more accurate the process; however, an oversized window may not only consume computational resources but also make too many errors in matching. In this paper, we proposed one method, CTHW, to increase the accuracy with a wavelet transform and another one, AWCT, to enable the conversion window size to be adjusted for every point. In the results of CTHW, only the bad matching is improved, which does not reduce the RMS and operation loading. We can see that the proposed CTHW can provide a better result with a small window size and be suitably applied to a system with low computational resources. AWCT further finds the number of edge points to select the suitable window size for each point. According to the results, AWCT achieves a better performance in reducing the operation times with acceptable quality. Compared with CT, its average reduction ratio of operation was found to be about 6.6%. When we applied the sparse census transform to AWCT, as AWSCT, and compared this with SCT, the average reduction ratio of operation was about 7.5%. In the future, it is worth studying the use of high-frequency information to improve the quality and reduce the operation, and further enhance the performance, of CT.

## Figures and Tables

**Table 1:** The results of CTHW and CT with percentages of bad matching pixels (PoBMP) (%).

Window Size	3 × 3		5 × 5		7 × 7		9 × 9		11 × 11		13 × 13	
Image Name	CT	CTHW	CT	CTHW	CT	CTHW	CT	CTHW	CT	CTHW	CT	CTHW
Moebius	78.75	41.95	53.97	24.17	39.35	21.33	31.72	19.85	27.34	19.36	24.66	19.40
Flowerpots	79.11	51.92	67.51	39.55	59.14	36.23	53.59	35.39	49.90	34.78	47.18	34.28
Reindeer	82.84	52.41	59.88	38.22	45.58	34.17	38.55	32.50	34.28	32.10	32.23	32.70
Cloth2	71.59	46.78	43.44	29.84	31.84	26.69	26.95	25.87	24.62	25.52	23.27	25.28
Midd1	84.97	70.29	68.38	54.44	58.33	46.47	53.21	41.88	50.12	38.82	48.17	37.10
Baby1	72.06	37.45	52.70	21.54	40.11	20.81	31.69	20.80	26.63	20.68	23.37	20.59

**Table 2:** Comparison of AWCT and CT.

Image Name	PoBMP of CT (21 × 21)	PoBMP of AWCT	RMS of CT (21 × 21)	RMS of AWCT	Reduction Ratio of Operation
**Moebius**	20.12	20.23	34.93	34.90	6.98%
**Flowerpots**	32.25	32.52	58.55	58.49	6.97%
**Reindeer**	29.55	29.42	47.48	47.49	6.11%
**Cloth2**	16.82	17.11	33.18	34.12	8.72%
**Midd1**	43.71	43.49	49.37	49.86	3.94%
**Baby1**	18.64	19.17	31.57	32.02	7.72%

**Table 3:** Comparison of AWSCT and SCT.

Image Name	PoBMP of SCT (21 × 21)	PoBMP of AWSCT	RMS of SCT (21 × 21)	RMS of AWSCT	Reduction Ratio of Operation
**Moebius**	25.00	25.82	38.81	39.48	6.16%
**Flowerpots**	39.06	39.80	61.80	61.98	8.08%
**Reindeer**	34.24	34.69	51.13	51.54	7.23%
**Cloth2**	22.36	23.18	43.86	45.09	9.42%
**Midd1**	48.33	48.89	52.99	53.24	5.03%
**Baby1**	24.89	26.24	36.49	37.33	9%
